# Practical Departments

**Published:** 1895-11-02

**Authors:** 


### PRACTICAL DEPARTMENTS.

### III.—DISINFECTION AND THE EXISTING DATA FOR ITS PRACTICE.

There are many obvious cases in which the application of heat for disinfection in whatever form is impossible, and the use of some chemical product is indicated. The extraordinary number of substances possessing, in greater or less degree, disinfectant or antiseptic properties, is a source rather of danger than of assistance to the medical officer. It is impossible to compare the disinfectant action of any process or substance without some control as to the resistance of the test organism used. The striking discrepancies which have appeared between the results of competent observers working ostensibly with the same organisms, on the same disinfectants, and in the same conditions, would be enough to show that wide variations of this sort must exist; and any doubt which there may have been on the point was settled by direct experiments long ago, in which various specimens of the same organism were exposed to the same disinfectant processes by the same experimenters with entirely contradictory results. There are many natural processes which make for disinfection. Extremely good results have been obtained in the disinfection of rooms by the combustion of a mere disinfectant *placebo* with a sufficiently disagreeable odour to ensure thorough ventilation of the room for three or four days. No person engaged in the work of disinfection is justified in neglecting to utilise any natural process such as this one of dilution of air-borne infection; but the chemical agents which may be employed in any systematic or scientific disinfection should be chosen for their direct germicidal power, and this can only be known by a most careful comparison of the most accurate and reliable experiments that have been made. For a large number of disinfectants which have been proposed, such experiment is not available; and the first maxim which persons engaged in disinfection should remember is to use no agent of which the effects have not been determined with sufficient accuracy.

The disinfection in bulk of sewage and of air are matters as to which, at present, sufficient data are scarcely available for formulating a satisfactory practice. In regard, however, to the important question of the disinfection of rooms much more is known. There can be no question that the use of sulphurous acid, whether produced by burning or used in a condensed form, is hopelessly unreliable. At the maximum percentage in which it can be applied it is insufficient to destroy many organisms, still more so to penetrate the slightest covering of dust or flock by which they may be protected. These results applying to rooms, whether wet or dry, have now been matters of scientific certainty for fifteen years; and in this respect, again, we find that England is almost alone in retaining a generally inconvenient, inefficient, and difficult process. The experiments of Professor Delépine in this country, and of Dr. Chamberland and others abroad, have drawn attention to the value of chlorinated lime. Further data are desirable in regard to the stability and application of this substance; but there is little doubt that for many purposes it will be an extremely powerful and convenient agent. At the same time its strong chemical reaction on any substances with which it may come in contact will make it useless for the disinfection of decorative surfaces, and of many materials which would both decompose and be injured by it. A broad question to be settled in regard to the disinfection of rooms is, for the moment, whether gases or vapours on the one hand should be used, or whether liquid disinfectants should be applied mechanically. Both systems have been largely tried, and the result is quite distinctly to show that disinfection by gases or vapours in any way yet suggested is either impracticable, unreliable, or both. In addition to these disadvantages, they have the objection of being in most cases extremely injurious to surfaces with which they come in contact, and of requiring for even such degree of accuracy as in the maximum they are capable of, a skill and care which it would be difficult for them to get, and which, in fact, they do not get. They yield a disinfectant atmosphere uncertain in composition; they fail to disinfect the air of rooms; and it cannot be doubted that, following the example, among others, of Sir Henry Littlejohn, of Edinburgh, and Sir C. Cameron, of Dublin, recourse will be had to the application of liquid disinfectants. In this method the only questions are as to the liquid to be chosen and the method of its application. There is some margin for choice of the liquid, and it would be beyond the scope of this article to recite the grounds on which one or other of the principal alternatives are to be preferred. It may, however, be broadly stated that the use of perchloride of mercury, one in a thousand, with a small percentage of common salt or tartaric acid, is not only theoretically efficient, but also has been tested in practice to an extent sufficient to confirm this view. It is probable, therefore, that for the time being this will be found the most convenient substance to be used for the disinfection of rooms when our sanitary authorities determine to abandon the use of fumigations. The method of application is a matter of more importance than is apparent at first sight. It was ascertained long ago that the process of washing often amounted to no more than the mechanical removing of organisms from one part of the surface to another, and that the process of drying, by which the disinfectant action is limited, is often too rapid to be consistent with disinfection. To meet this difficulty the Equifex spray process is probably the best means available. It consists in projecting on to the surface to be disinfected an extremely fine spray or cloud emitted at high velocity from a nozzle. The fineness of the spray prevents the liquid from pouring down the walls, and not only avoids the spoiling of delicate surfaces, but also materially increases the time required for drying; so that the disinfectant action is more prolonged than with washing. The saturation of the neighbouring atmosphere by such part of the spray as has not struck the walls must certainly not be taken to effect real disinfection of the air, although it does a good deal towards it, and, possibly, may ultimately be found to really disinfect it. For the time being, however, it must be considered that in this process, as in those of fumigation and of washing, the only reliable disinfection of air is its dilution by ventilation. The effect of the spray process has been observed with special care in the municipality of Paris. Prior to its adoption the process used was that of sulphur fumigation, and in a recent debate in the Academy of Medicine of Paris it was shown that one of the great troubles of the sanitary service was the liability to re-infection in houses disinfected by this means. Since, however, this spray process has been substituted, involving the application of a disinfectant of known value, and assuring its distribution all over the infected surfaces, these re-infections and house epidemics have disappeared. The fact is the more notable because the lamented sanitarian who drew the attention of the Academy to the fact had, in earlier days, been a strong advocate of the fumigation system. The alternative method of dry bread is not likely to find favour over here, nor has it been generally adopted abroad. Those surfaces which cannot stand heat must be sprayed. The disinfectant chosen must be one as to the efficiency of which reliable data exist, and it should not be changed without the authority of accurate comparative experiments, in which the same organisms are simultaneously offered to the action of the new and the old disinfectant. In this way the practice of house disinfection will be placed on a sound foundation, worthy to rank with saturated steam. The difficulty which is felt by many authorities of providing accommodation for inmates of rooms during the twenty-four hours' farce which is at present played with sulphurous acid, will be overcome without expense and with a gain to the efficiency of the service.

The data which have been quoted above are given not by any means as exhausting the vast subject to which they relate. The more carefully it is considered, the more it will be seen that an immense labour would be involved in a complete treatment of the subject, and it may be long before such treatment is obtained. The facts which have been stated are those on which a reliable practice embodying the best ascertained results may be constructed. We cannot afford to neglect any alternative means which may be likely to offer advantage. It is, however, still more desirable that reliable means having once been adopted, strict and accurate investigation should be made before they are changed.

